# Revealing the most common reporting errors through data mining of the report proofreading process

**DOI:** 10.1007/s00330-020-07306-6

**Published:** 2020-09-30

**Authors:** Jan Vosshenrich, Ivan Nesic, Joshy Cyriac, Daniel T. Boll, Elmar M. Merkle, Tobias Heye

**Affiliations:** Department of Radiology, University Hospital Basel, Petersgraben 4, 4031 Basel, Switzerland

**Keywords:** Report, Education, Residency, Medical errors, Patient safety

## Abstract

**Objectives:**

To investigate the most common errors in residents’ preliminary reports, if structured reporting impacts error types and frequencies, and to identify possible implications for resident education and patient safety.

**Material and methods:**

Changes in report content were tracked by a report comparison tool on a word level and extracted for 78,625 radiology reports dictated from September 2017 to December 2018 in our department. Following data aggregation according to word stems and stratification by subspecialty (e.g., neuroradiology) and imaging modality, frequencies of additions/deletions were analyzed for findings and impression report section separately and compared between subgroups.

**Results:**

Overall modifications per report averaged 4.1 words, with demonstrably higher amounts of changes for cross-sectional imaging (CT: 6.4; MRI: 6.7) than non-cross-sectional imaging (radiographs: 0.2; ultrasound: 2.8). The four most frequently changed words (right, left, one, and none) remained almost similar among all subgroups (range: 0.072–0.117 per report; once every 9–14 reports). Albeit representing only 0.02% of analyzed words, they accounted for up to 9.7% of all observed changes. Subspecialties solely using structured reporting had substantially lower change ratios in the findings report section (mean: 0.2 per report) compared with prose-style reporting subspecialties (mean: 2.0). Relative frequencies of the most changed words remained unchanged.

**Conclusion:**

Residents’ most common reporting errors in all subspecialties and modalities are laterality discriminator confusions (left/right) and unnoticed descriptor misregistration by speech recognition (one/none). Structured reporting reduces overall error rates, but does not affect occurrence of the most common errors. Increased error awareness and measures improving report correctness and ensuring patient safety are required.

**Key Points:**

*• The two most common reporting errors in residents’ preliminary reports are laterality discriminator confusions (left/right) and unnoticed descriptor misregistration by speech recognition (one/none).*

*• Structured reporting reduces the overall the error frequency in the findings report section by a factor of 10 (structured reporting: mean 0.2 per report; prose-style reporting: 2.0) but does not affect the occurrence of the two major errors.*

*• Staff radiologist review behavior noticeably differs between radiology subspecialties.*

**Electronic supplementary material:**

The online version of this article (10.1007/s00330-020-07306-6) contains supplementary material, which is available to authorized users.

## Introduction

Every radiology residency program is built on several backbones to provide residents with the necessary skills to ultimately function as an independent radiologist. These pillars include knowledge of the radiological appearance of diseases, technical expertise to perform appropriate diagnostic tests, and communication skills to transmit information on imaging studies to referring physicians and patients. While both diagnostic and technical skills are continuously trained during image interpretation, case review with attendings and collaboration with radiology technologists, communication skills, and especially the radiology report creation is not always a main focus in resident education.

Given that the radiology report is the radiologist’s main communication tool to transfer information, training in report writing is crucial to provide residents with the abilities to act as reputable partners for clinicians. Furthermore, and in contrast to many other medical specialties, radiologists’ reporting errors are easily traceable, may it be speech recognition errors, overlooked findings, or even confusions of laterality discriminators. Finally, the radiology report is a legal document and may be used in medical malpractice claims.

During radiology residency, residents’ reports are signed-off by a staff radiologist to ensure accuracy. This review process serves to identify and correct many different types of errors. However, the amount and method of feedback a resident receives concerning his reporting style and accuracy varies among institutions and attendings. Currently, there is no standardized way to track the report correction process. Only a few customized tools tracking changes between residents’ preliminary reports and documents finalized by staff radiologists fill this gap [1–5]. Also, our department introduced a custom-developed report comparison tool in 2017 to facilitate individual feedback to residents concerning changes made to their preliminary reports.

The purpose of this study was to assess the most common reporting errors in residents’ preliminary reports as well as variation in type and amount of errors between different reporting standards (structured vs. non-structured) based on data mining of changes from the report proofreading process on a word level to understand recurring errors and derive possible educational implications.

## Materials and methods

Institutional review board approval and the requirement for informed consent was waived, since no patient identifiers were used in any part of our retrospective study. Data solely consisted of plain text from radiology reports created in our department, which could neither be tracked back to individual patients nor radiologists.

### Data acquisition

In 2017, a custom-developed report comparison tool was introduced in our tertiary care radiology department to help residents track changes made to their preliminary reports by staff radiologists during sign-off. It automatically queries the content of all reports every 15 min from the institutional radiological information system (RIS). Tracking of report content changes is based on different states of a document along its workflow pathway within the RIS. Our RIS (Centricity^TM^ RIS-i 6.0, GE Healthcare) distinguishes between the following report states: written, preliminary, and approved.

**Fig. 1:**
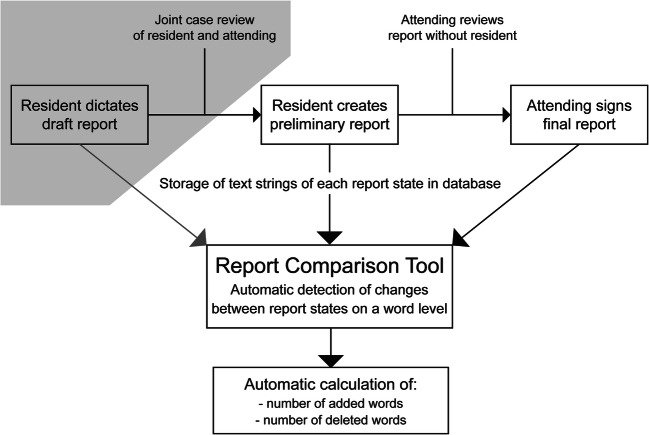
Flowchart of the radiology report pathway in our department from initial draft to final report, including the report review process and data storage by the report comparison tool queries. Of note: draft reports (gray overlay in the flowchart) are also tracked by the tool but were not included into the present study

After dictating an initial written report using SpeechMagic 8 software (Nuance) and joint case review with an attending, the resident sends a corrected preliminary report to the respective staff radiologist for proofreading and editing. By signing the corrected report, its status changes to approved (Fig. 1).

**Fig. 2:**
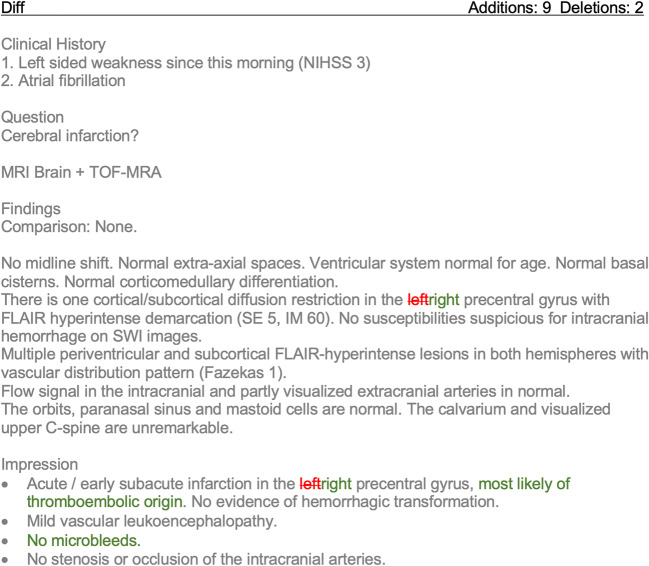
Screenshot of the “difference view” in our report comparison tool. Tracked changes between preliminary and final report are visualized in a color-coded fashion (red for deletions and green for additions) to allow easy identification by the user. Of note: report was translated into English language

The report comparison tool visualizes staff radiologists’ edits on the latest version of a resident’s preliminary report through color coding (Fig. 2). Additions and deletions can be extracted on a word level.

Reports approved directly without prior saving as written or preliminary report (i.e., reports dictated and immediately signed by attendings) are not tracked due to lack of different report states.

### Data

**Fig. 3:**
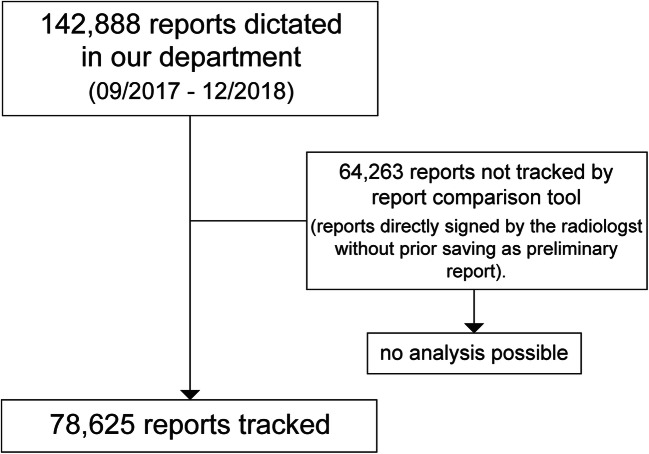
Flowchart of the data gathering process for the report comparison dataset

A total of 142,888 reports were created from 1st of September 2017 to 31st of December 2018 in our department. A total of 78,625 were tracked by the report comparison tool and available for analysis (Fig. 3). Data of all subspecialty sections (neuroradiology, musculoskeletal imaging, cardiothoracic imaging, body imaging, breast imaging, and nuclear medicine) and imaging modalities (radiographs, CT, MRI, ultrasound, mammography, scintigraphy, SPECT, and PET-CT) were analyzed without preselection. Furthermore, it was noted if subspecialties were using structured reporting (body and cardiothoracic imaging) or reported in prose style (all other sections). Body and cardiothoracic imaging subspecialties report all examinations without exception using structured templates, either containing subheadings for body regions and distinct organs with prepopulated normal findings (e.g., CT abdomen/pelvis) or checklists for standardized reporting of features (e.g., rectal cancer staging MRI).

### Data analysis

Added and deleted words from residents’ preliminary reports were extracted from the report comparison tool along with the following metadata: findings or impression report section, subspecialty, and imaging modality and stored in a data table. Stop words, such as be, as, the, a, and an, were excluded because they didn’t convey information. Each row of the data tables thus represented a quantitative evaluation for a specific word added or deleted from reports of a certain imaging modality within a distinct subspecialty (e.g., the word bleeding was deleted 100 times from the findings section of MRI reports in neuroradiology).

Subsequently, words were aggregated according to their word stem (lexeme). A lexeme is a set of forms taken by a single root word (lemma). The root word is the citation form. In the English language, this process may not seem particularly necessary; however, our study is based on reports in German language. Conditioned by its grammatical complexity, this step was important to gain realistic word counts, since a word’s form differs according to grammatical gender, number, and case. As an example, the word right (rechts in German) may take the form of rechte, rechten, rechtem, rechter, and rechtes. If only considering the lemma rechts, and ignoring all other forms, word counts would not have been accurate.

The total number of additions or deletions of each lexeme was calculated separately for findings and impression report sections, each subspecialty and imaging modality. Finally, rankings of most frequently added and deleted words were created. For breast imaging and nuclear medicine, reports of available modalities were aggregated. In breast imaging, the reporting routine made this step necessary, as often patients get a mammography and a sonography in one appointment which are dictated together in one single report. In nuclear medicine, this step was necessary to gather a sufficiently large number of reports for analysis.

### Mathematical and graphical analysis

Sums of additions, deletions, and report numbers as well as rankings were analyzed and graphically visualized using commercially available software (JMP® 14.0, SAS Institute Inc.). Ratios of additions and deletions per report section, imaging modality, and subspecialty section were calculated. Relative and absolute differences between the ratios of all subgroups were assessed. Finally, variations between reporting standards were investigated.

## Results

**Fig. 4:**
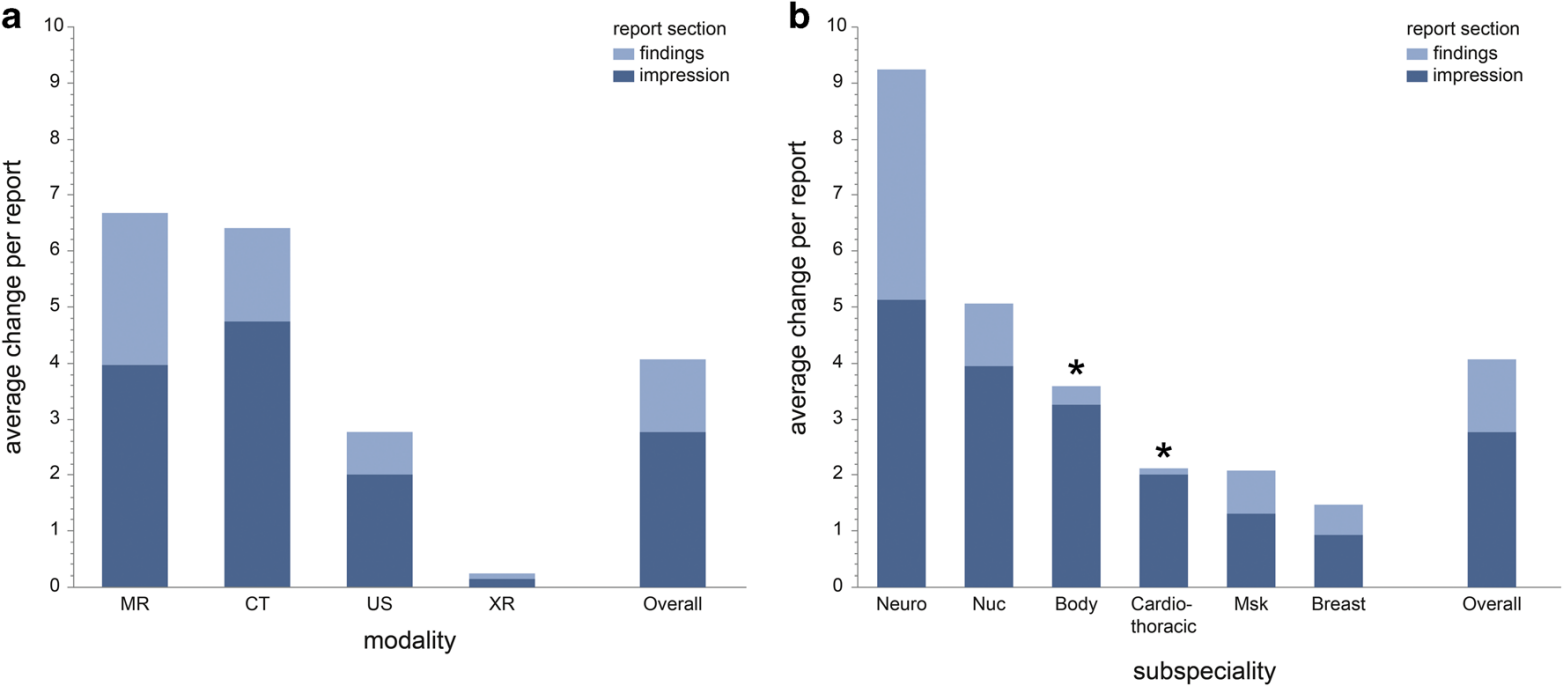
Bar graphs depicting the average changes per report (*y*-axis) for the distinct subspecialty sections (**a**; *x*-axis) and imaging modalities (**b**; *x*-axis). The asterisk (*) indicates subspecialty sections solely using structured reports. Please note the substantially lower average changes per report in the findings section for structured reporting in body imaging compared with prose-style reporting neuroradiology, while both predominantly report cross-sectional imaging studies. Similarly, there are noticeably lower average changes per report in the findings section for structured reporting in cardiothoracic imaging compared with prose style in musculoskeletal imaging, while both reporting a large number of radiographs. Neuro = neuroradiology; Nuc = nuclear medicine; body = body imaging; cardiothoracic = cardiothoracic imaging; Msk = musculoskeletal imaging; breast = breast imaging

The total numbers of reports subspecialties and imaging modalities contributed are listed in Table 1. On average, final reports consisted of 131.1 words (radiographs: 50.6; ultrasound: 131.0; CT: 195.8 and MRI: 134.4). Overall, we found 2.2 additions (174,760 words) and 1.9 deletions (146,012 words), or 4.1 changes (320,772 words) per report. Change ratios in the findings report section were lower with 0.6 additions and 0.8 deletions per report compared with the impression section with 1.7 additions and 1.1 deletions on average. Ratios for all subspecialties and imaging modalities are listed in Table 2 and graphically presented in Fig. 4.

**Table 1:** Total number of reports analyzed per imaging modality and subspecialty section. Percentages in brackets represent fraction of total. * = for breast imaging and nuclear medicine sections, different modalities were added and analyzed together (breast imaging: mammography, ultrasound; nuclear medicine: PET-CT, SPECT and scintigraphy)

Modality	Radiology subspecialty section						
	Body imaging *N* (% of total)	Cardiothoracic imaging *N* (% of total)	Musculoskeletal imaging *N* (% of total)	Neuroradiology *N* (% of total)	Breast imaging* *N* (% of total)	Nuclear medicine* *N* (% of total)	Total *N* (% of total)
Radiography/fluoroscopy	577 (0.7)	9527 (12.1)	14,064 (17.9)	526 (0.7)	–	–	24,694 (31.4)
Ultrasound	1761 (2.2)	3 (0.0)	263 (0.3)	12 (0.0)	–	–	2039 (2.6)
CT	6952 (8.8)	8114 (10.3)	2020 (2.6)	7774 (9.9)	–	–	24,860 (31.6)
MRI	2404 (3.1)	1255 (1.6)	5484 (7.0)	9647 (12.3)	–	–	18,790 (23.9)
Mammography*	–	–	–	–	3902 (5.0)	–	3902 (5.0)
Other*	–	–	–	–	–	4310 (5.5)	4310 (5.5)
Total	11,694 (14.8)	18,929 (24.0)	21,831 (27.8)	17,959 (22.9)	3902 (5.0)	4310 (5.5)	78,625 (100)

**Table 2:** Overview of total counts of additions and deletions to both findings and impression section of all of analyzed radiology reports. Numbers in brackets represent corresponding fraction of total additions or deletions in the respective section of reports and ratio of changes per report in the respective imaging section or modality. Changes to findings section of reports in subspecialties using structured reporting are marked in italics

Total additions and deletions to preliminary radiologic reports						
Section or modality	Findings section			Impression section		
	Additions *N* (% of total; changes/report)	Deletions *N* (% of total; changes/report)	Total changes *N* (% of total; changes/report)	Additions *N* (% of total; changes/report)	Deletions *N* (% of total; changes/report)	Total changes *N* (% of total; changes/report)
Body imaging	2597 (6.0%; *0.22*)	1225 (2.1%; *0.11*)	3822 (3.7%; *0.33*)	22,738 (17.3%; 1.94)	15,436 (17.9%; 1.32)	38,174 (17.5%; 3.26)
Cardiothoracic imaging	1254 (2.9%; *0.07*)	838 (1.4%; *0.04*)	2092 (2.0%; *0.11*)	23,734 (18.1%; 1.25)	14,287 (16,6%; 0.76)	38,021 (17.5%; 2.01)
Musculoskeletal	9125 (21.1%; 0.42)	7580 (12,7%; 0.35)	16,705 (16.2%; 0.77)	16,594 (12.6%; 0.76)	12,024 (14.0%; 0.55)	28,618 (13.2%; 1.31)
Neuroradiology	26,664 (61.5%; 1.49)	47,110 (78.7%; 2.62)	73,774 (71.5%; 4.11)	54,598 (41.5%; 3.04)	37,504 (43.6%; 2.09)	92,102 (42.3%; 5.13)
Breast imaging	1239 (2.9%; 0.32)	845 (1.4%; 0.22)	2084 (2.0%; 0.54)	2478 (1.9%; 0.64)	1133 (1,3%; 0.29)	3611 (1.7%; 0.93)
Nuclear medicine	2447 (5.7%; 0.57)	2317 (3.9%; 0.54)	4764 (4.6%; 1.11)	11,292 (8.6%; 2.62)	5733 (6.7%; 1.33)	17,025 (7.8%; 3.95)
CT	18,003 (41.6%; 0.72)	23,700 (39.6%; 0.95)	41,703 (40.4%; 1.67)	71,842 (54.4%; 2.89)	46,013 (53.4%; 1.85)	117,855 (54.2%; 4.74)
MRI	19,062 (44.0%; 1.02)	31,773 (53.1%; 1.69)	50,835 (49.2%; 2.71)	44,842 (34.0%; 2.39)	29,725 (34.5%; 1.58)	74,567 (34.3%; 3.97)
Ultrasound	1249 (2.9%; 0.61)	309 (0.5%; 0.15)	1558 (1.5%; 0.76)	2224 (1.7%; 1.09)	1874 (2.2%; 0.92)	4098 (1.9%; 2.01)
Radiography/fluoroscopy	1326 (3.1%; 0.05)	971 (1.6%; 0.04)	2297 (2.2%; 0.09)	2071 (1.6%; 0.08)	1639 (2.0%; 0.07)	3710 (1.7%; 0.15)
Overall	43,326 (100%; 0.55)	59,895 (100%; 0.75)	103,221 (100%; 1.30)	131,434 (100%; 1.67)	86,117 (100%; 1.10)	217,551 (100%; 2.77)

### Most frequently changed words

Both in the findings and impression report section, the words “one, none, right, and left” always represented the most frequently added and deleted words overall. Changes of these words occurred 0.097 (one), 0.117 (none), 0.074 (right), and 0.072 (left) times per report, or on average once every 9–14 reports. Analysis of subspecialties and imaging modalities confirmed this observation, as these four words were among the most frequent additions and deletions in almost every subgroup. The reporting standard had no effect on change frequencies of these words.

Only in a few instances, e.g., in the findings section of breast imaging reports, other words were changed more often. Frequency tables for the most frequently added words to findings and impression report sections are listed in Tables 3 and 4. Similar tables for deleted words can be found in the supplement.

**Table 3:** Overview of the top 5 most frequently added words to the findings section of analyzed radiology reports. Numbers in brackets represent count of additions and corresponding fraction of total additions in the respective subspecialty section or imaging modality. The last row represents results for all analyzed reports overall. The four most frequently added words overall (one, none, left, right) are marked in italics

Top 5 most frequently added words to findings section of reports						
Section or modality	1st word (*N*; % of total)	2nd word (*N*; % of total)	3rd word (*N*; % of total)	4th word (*N*; % of total)	5th word (*N*; % of total)	Sum *N* (% of total)
Body imaging	*None* *(103; 4.0%)*	Normal (69; 2.7%)	*Left* *(45; 1.7%)*	*One* *(37; 1.4%)*	*Right* *(36; 1.4%)*	290 (11.2%)
Cardiothoracic imaging	*None* *(36; 2.9%)*	*Left* *(25; 2.0%)*	*One* *(16; 1.3%)*	Aorta (16; 1.3%)	*Right* *(14; 1.1%)*	107 (8.6%)
Musculoskeletal	*None* *(220; 2.4%)*	Mild (112; 1.2%)	*One* *(111; 1.2%)*	*Right* *(93; 1.0%)*	As well as (87; 1.0%)	623 (6.8%)
Neuroradiology	*None* *(817; 3.3%)*	*Right* *(626; 2.4%)*	*Left* *(528; 2.0%)*	Bilateral (372; 1.4%)	*One* *(356; 1.3%)*	2753 (10.4%)
Breast imaging	*None* *(36; 2.9%)*	Mammography (26; 2.1%)	Sonography (20; 1.6%)	*One* *(16; 1.3%)*	*Left* *(9; 0.7%)*	107 (8.6%)
Nuclear medicine	*Right* *(64; 2.6%)*	*Left* *(58; 2.4%)*	*None* *(57; 2.3%)*	*One* *(37; 1.5%)*	Nodule (31; 1.3%)	247 (10.1%)
CT	*None* *(612; 3.4%)*	*Right* *(396; 2.2%)*	*Left* *(359; 2.0%)*	Bilateral (235; 1.3%)	*One* *(209; 1.2%)*	1811 (10.1%)
MRI	*None* *(551; 2.9%)*	*Right* *(314; 1.7%)*	*Left* *(298; 1.6%)*	*One* *(273; 1.4%)*	Normal (216; 1.1%)	1652 (8.7%)
Ultrasound	Normal (56; 2.6%)	*None* *(43; 3.4%)*	*One* *(24; 1.9%)*	Or (22; 1.8%)	*Right* *(18; 1.4%)*	163 (11.1%)
Radiography/fluoroscopy	*None* *(26; 2.0%)*	*Right* *(18; 1.4%)*	Bilateral (16; 1.2%)	*Left* *(14; 1.1%)*	*One* *(14; 1.0%)*	88 (6.7%)
Overall	*None* *(1325; 3.1%)*	*Right* *(814; 1.9%)*	*Left* *(755; 1.7%)*	*One* *(573; 1.3%)*	Normal (414; 1.0%)	3881 (9.0%)

**Table 4:** Overview of the top 5 most frequently added words to the impression section of analyzed radiology reports. Numbers in brackets represent count of additions and corresponding fraction of total additions in the respective subspecialty section or imaging modality. The last row represents results for all analyzed reports overall. The four most frequently deleted words overall (one, none, left, right) are marked in italics

Top 5 most frequently added words to impression section of reports						
Section or Modality	1st word (*N*; % of total)	2nd word (*N*; % of total)	3rd word (*N*; % of total)	4th word (*N*; % of total)	5th word (*N*; % of total)	Sum *N* (% of total)
Body imaging	*One* *(717; 3.2%)*	*None* *(544; 2.4%)*	*Left* *(293; 1.3%)*	*Right* *(287; 1.3%)*	Recommended (264; 1.2%)	2105 (9.4%)
Cardiothoracic imaging	*One* *(630; 2.2%)*	*Right* *(419; 1.8%)*	*None* *(411; 1.7%)*	*Left* *(378; 1.6%)*	Pulmonary (227; 1.0%)	2065 (8.8%)
Musculoskeletal	*None* *(325; 2.0%)*	*One* *(298; 1.8%)*	*Right* *(218; 1.3%)*	*Left* *(208; 1.3%)*	As well as (192; 1.2%)	1241 (7.6%)
Neuroradiology	*None* *(1717; 3.1%)*	*One* *(1220; 2.2%)*	*Left* *(1186; 2.2%)*	*Right* *(1164; 2.1%)*	Bleeding (531; 1.0%)	5818 (10.6%)
Breast imaging	*Left* *(109; 4.4%)*	*Right* *(105; 4.2%)*	*None* *(82; 3.3%)*	*One* *(60; 2.4%)*	Breast (43; 1.7%)	399 (16.0%)
Nuclear medicine	*One* *(398; 3.5%)*	Bone Metabolism (226; 2%)	*Right* *(213; 1.9%)*	*Left* *(204; 1.8%)*	*None* *(137; 1.2%)*	1178 (10.4%)
CT	*One* *(1797; 2.5%)*	*None* *(1589; 2.2%)*	*Right* *(1389; 1.9%)*	*Left* *(1357; 1.9%)*	Recommended (588; 0.8%)	6720 (9.3%)
MRI	*None* *(1283; 2.9%)*	*One* *(1035; 2.3%)*	*Left* *(728; 1.6%)*	*Right* *(714; 1.6%)*	Lesion (545; 1.2%)	4305 (9.6%)
Ultrasound	*None* *(105; 4.7%)*	*One* *(93; 4.2%)*	Evidence (33; 1.5%)	Not (27; 1.2%)	Without (27; 1.2%)	285 (12.8%)
Radiography/fluoroscopy	*One* *(58; 2.8%)*	*None* *(52; 2.5%)*	Additional (22; 1.1%)	*Right* *(21; 1.0%)*	Evidence (19; 1.0%)	172 (8.4%)
Overall	*One* *(3323; 2.5%)*	*None* *(3216; 2.5%)*	*Right* *(2406; 1.8%)*	*Left* *(2387; 1.8%)*	Or (1148; 0.9%)	12,480 (9.5%)

The aforementioned four words accounted for 8.0% to 9.7% of the total number of additions or deletions in the distinct report sections, meaning that e.g. 0.02% of all distinct words (4 of 19,746 words) were responsible for almost 10% of all deletions from the impression section.

**Fig. 5:**
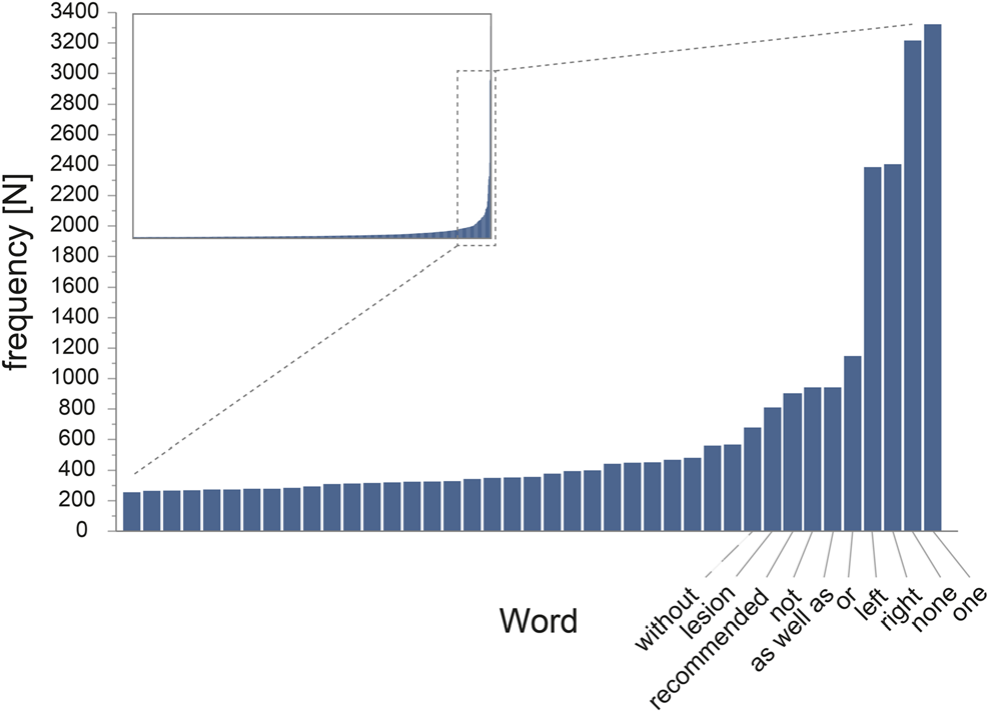
Bar graph depicting the distribution of frequencies for each unique added word to the impression section of all reports. Each bar on the *x*-axis represents a distinct word, the height of each bar (*y*-axis) the frequency of additions. Data is exponentially distributed: only few words were added frequently (right side of the graph), while a large number of words was only added a few times (long flat portion of the graph on the left side). Inset shows entire dataset, main graph depicts only most frequently added words. Of note: translations of the four most frequently changed words in German (reporting language) are (“one” = “eine”; “none” = “keine”; “left” = “links”; “right” = “rechts”)

Both graphically and numerical, a sharp transition in addition/deletion frequencies was seen following the words none, one, right, and left. For instance, these four words represented 8.6% of all additions (11,332 of 131,434) to the impression section of reports, a fraction twice as high compared with the remaining six words in the top ten frequency ranking (4.1%; 5419 of 131,434). When plotting distinct words and change frequencies graphically, distribution was exponential with a large amount of distinct words with low addition/deletion counts and a small amount of words with high addition/deletion counts (Fig. 5).

### Analysis of report sections and reporting standard

The number of modifications in the findings section was substantially lower compared with the impression section for all subspecialties and imaging modalities, with an overall ratio of 1.3 words per report in the findings section vs. 2.8 in the impression section. Largest net difference of ratios between report sections was observed in body imaging with 3.0 (0.3 changes per report in the findings vs. 3.3 in the impression section).

In the two subspecialties employing structured reporting, change ratios in the findings section were noticeably lower with a mean of 0.2 per report (0.1 in cardiothoracic and 0.3 in body imaging) compared with subspecialties reporting in prose style (mean: 2.0, range: 0.5–4.1). This was observed for both sections with high amounts of cross-sectional imaging (e.g., 0.3 per report in body imaging vs. 4.1 in neuroradiology), and subspecialties with large volumes of radiographs (e.g., 0.1 in cardiothoracic vs 0.8 in musculoskeletal imaging). For the impression report section, no such differences were noted (2.0–3.3 for structured reporting vs. 0.9–5.1 for prose reporting).

When comparing change ratios per report section proportionally, structured reports showed an eleven- and twenty-fold lower amount of changes in the findings than in the impression section (0.3 vs. 3.3 per report in body imaging and 0.1 vs. 2.0 in cardiothoracic imaging), respectively. For prose-style reports, proportional differences were markedly less pronounced, ranging from 1.3-fold (neuroradiology, 4.1 vs. 5.1) to 3.6-fold (nuclear medicine, 1.1 vs. 4.0).

### Subspecialty section analysis

The highest amount of modifications was seen in neuroradiology with 9.2 changes per report (4.1 in the findings and 5.1 in the impression section). The neuroradiology changes comprised 71.5% (findings section) and 42.3% (impression section) of all changes in the datasets, while the number of reports in this subspecialty only represented 22.9% of data. In contrast, musculoskeletal imaging contributed 27.8% of reports; however, only 16.2% (findings section) and 13.2% (impression section) of changes were conducted in this section.

The lowest change ratio was noted in breast imaging (1.5). The other subspecialty sections ranged from 2.1 (musculoskeletal and cardiothoracic imaging) to 5.1 (nuclear medicine) per report (Fig. 4).

### Imaging modality analysis

Overall change ratios were higher for cross-sectional imaging (CT: 6.4 per report; MRI: 6.7) compared with radiographs (0.3) and ultrasound exams (2.8).

A total of 91.2% of all changes to the findings and 88.5% of changes to the impression section occurred in CT and MRI reports. However, these two modalities combined only represented 55.5% of reports in the dataset. In contrast, the number of modifications to reports of radiographs was small with 2.2% of total changes in the findings and 1.7% of total changes in the impression section, while the fraction of total number of reports was 31.4%.

## Discussion

The aim of our study was to analyze the most common errors in residents’ preliminary reports corrected during report proofreading. The most frequently changed words (one, none, right, and left) remained almost identical, irrespective of subspecialty, imaging modality, or reporting standard, even though overall error frequencies were lower for structured reporting. This suggests fundamental and systematic errors which are not limited to specific exams and need to be addressed in residents’ education and radiology practice in general.

Overall, the amount of modifications to residents’ reports in our department seems to be low with a median of 4.1 changed words per report, considering the average report length of 131.1 words. However, we did not find any study we could compare our results to. Substantially higher change ratios for cross-sectional imaging studies are likely attributed to their complexity. This conclusion is also supported by higher change ratios in subspecialties with high volumes of CT and MRI examinations. Subspecialties with large amounts of radiographs, where reports are supposedly shorter and imaging studies easier to interpret, had demonstrably lower change ratios. Other explanations may be greater emphasis of attendings when reviewing cross-sectional imaging studies and differences in proofreading behavior, since especially neuroradiologists’ change ratios surpassed those of all other subspecialties.

Change ratios in the findings report section were substantially lower in subspecialties using structured reporting. Differences in impression section ratios were much less pronounced between reporting standards. We attribute this to the fact, that the impression is dictated in prose style in all subspecialties to enable transmission of unambiguous conclusions, so clinicians can adapt patient-management. This may not be achieved with predefined templates, since imaging findings, although presented in a structured manner, need to be put in the right context for the individual patient. The resulting large proportional differences between report sections (low change ratios in the findings vs. high change ratios in the impression section) therefore demonstrate the benefits of structured reporting, i.e., reduction of errors and thus less corrections required by staff radiologists, who can now put more emphasis on optimal wording of the impressions. Several existing studies support this conclusion, showing that benefits of structured reporting are higher diagnostic accuracy and lesser missed findings and orthographic errors [6–9].

Change ratios of the four most frequently added/deleted words were much higher than for any other word. They remained similar, irrespective of subspecialty and imaging modality. High counts of additions/deletion of the words right and left can to a certain extent be explained by missed or over-read findings in the initial report, being added or deleted during case review by staff radiologists. However, a substantial amount of modifications must be attributed to laterality discriminator confusions which were substituted during report proofreading. Several studies tried to quantify the amount of this error [10, 11]; however, they are based on finalized reports or report addendums. Our study in contrast used residents’ preliminary reports. This explains why our observed laterality discriminator change frequencies (once every 14 reports) were more than 100-times higher than previously reported error rates, ranging from 0.048 to 0.055% of reports (equaling 1/1818 to 1/2083 reports) [11, 12]. This underlines the importance of case review by attendings, who regularly seem to prevent this error from appearing in final reports.

A possible explanation for frequent left/right confusions may be that review of imaging studies is initially counterintuitive for residents, who have to describe pathologies from a patient’s view and not their visual perspective. Factors like stress, fatigue, distractions, and time-pressure further increase the likelihood of left/right confusions, as they do in other medical fields [13]. They predestine for higher error rates compared with the normal population, where survey-based studies already found up to one third of adults experiencing laterality discrimination confusions in daily life [14, 15].

Several custom-developed software solutions aiming to decrease this error type have been devised. These include color-coded laterality discriminator crosschecks prior to report signing [16] and algorithms comparing report content to patients’ Health Level 7 metadata [17]. However, no software solution is available to detect discrepancies between findings and impression report sections in real time. With the current rapid progress in artificial intelligence and natural language processing, this issue may be addressed in the near future.

The high change ratios of the words one and none in our study are again at least partly conditioned by content being added or deleted during proofreading. However, a large portion of modifications likely resulted from speech recognition errors being substituted by staff radiologists. The words “one” and “none” translate as “eine” and “keine” in German, demonstrating that the descriptors are prone to misregistration in both languages. However, no previous study investigated this particular error type for the speech recognition software we use at our institution. Similar to laterality discriminators, descriptor errors in radiology reports can cause misunderstandings and are potentially harmful for patients.

Speech recognition errors are well known since the introduction of this technique and are more common compared with manual report transcription [18]. Errors vary in importance, ranging from trivial spelling errors to alterations of meaning and possible interpretation of reports [19]. This affects all medical subspecialties using speech recognition with reported overall error rates of up to 7.4% [20]. Even though speech recognition solutions improved over the years, error detection still solely depends on proofreading. This fact supports the need for systematic error analysis and software tools assisting in this process. A first step in this direction was taken by the growing number of custom-developed report comparison tools [1–5]. They facilitate the review process, providing residents with individual feedback on recurring errors and help attendings to convey teaching points when joint case review is impossible. Besides providing feedback, report comparison can be used to review any error type on a word level. When aggregating data from individual users, as we demonstrated, meta-level datasets provide insights into common errors or undesired reporting habits needing to be addressed in teaching sessions.

Our study has several limitations. It is based on reports in German language; however, since we took grammatical particularities into account, we think our results are transferable to other languages. The analysis is based on a moderately sized data sample from a single tertiary care university hospital; nevertheless, data should be sufficient to draw conclusions with regard to major reporting errors and impact of structured reporting. Also, to our knowledge, there is no similar study in the literature we could compare our results with. Our investigation solely based on the counts of added and deleted words during report proofreading. Data thus not only includes correction of errors but likely also contains text shifts between report sections and content which was added during proofreading if important information was missing in preliminary reports. This may have exaggerated counts of additions and deletions. Nevertheless, the methodology we used should be reproducible in other radiology department setups to allow for future comparison of our results.

In conclusion, we demonstrated that the most frequent errors are laterality discriminator confusions and descriptor misregistration by speech recognition, remaining similar among all modalities and subspecialties. The implementation of structured reporting templates can reduce overall error rates, but does not affect the two major errors types. As both errors have potential implications for patient safety, teaching measures need to be taken to help avoid these errors in the future. These include regular teaching sessions for residents, especially to raise awareness in new junior residents joining a program, and elaboration of existing software solutions, such as report comparison tools, with additional features (e.g., top ten rankings of own mistakes), to foster understanding of errors and strategies to avoid these.

## Electronic supplementary material

ESM 1 (PDF 43 kb)

## Abbreviations

CT: Computed tomography
MRI: Magnetic resonance imaging
PET-CT: Positron emission tomography–computed tomography
RIS: Radiology information system
SPECT: Single-photon emission computed tomography
