# Supplementary material for: Revealing the most common reporting errors through data mining of the report proofreading process
Source: Eur Radiol. 2020 Sep 30;31(4):2115–25. doi: 10.1007/s00330-020-07306-6 (PMC7979672; doi:10.1007/s00330-020-07306-6)
Supplement: Supplementary file 1 — (PDF 43 kb) [file 330_2020_7306_MOESM1_ESM.pdf]

Supplementary Material

Table 5 Overview of the top 5 most frequently deleted words from the findings section of analyzed radiology reports. Numbers in brackets represent count of deletions and corresponding fraction of total deletions in the respective subspecialty section or imaging modality. The last row represents results for all analyzed reports overall. The four most frequently added words overall (one, none, left, right) are marked in bold.

Top 5 Most Frequently Deleted Words from Findings Section of Reports

**Section or Modality**	**1st** word (N; % of total)	**2nd** word (N; % of total)	**3rd** word (N; % of total)	**4th** word (N; % of total)	**5th** word (N; % of total)	**Sum** N (% of total)
Body imaging	**None (76; 6.2%)**	**Left (24; 2.0%)**	**Right (15; 1.2%)**	Free (11; 0.9%)	Fluid (11; 0.9%)	137 (11.2%)
Cardiothoracic imaging	**None (64; 7.6%)**	**Right (20; 2.4%)**	**Left (10; 1.2%)**	**One (10; 1.2%)**	Pleural Effusion (9; 1.1%)	112 (13.5%)
Musculoskeletal	**None (300; 4.0%)**	**One (122; 1.6%)**	Normal (103; 1.4%)	Mild (98; 1.3%)	Unremarkable (81; 1.1%)	704 (9.4%)
Neuroradiology	**None (1,664; 3.5%)**	**Right (1,157; 2.5%)**	**Left (1,110; 2.4%)**	**One (883; 1.9%)**	Normal (760; 1.6%)	5,554 (11.9%)
Breast Imaging	**None (35; 4.2%)**	**Right (19; 2.3%)**	Bilateral (19; 2.3%)	Axillary (18; 2.2%)	Lymph node (18; 2.2%)	109 (13.2%)
Nuclear medicine	**Right (65; 2.8%)**	**Left (62; 2.7%)**	Volume (51; 2.2%)	**None (40; 1.7%)**	Thyroid Lobe (35; 1.5%)	253 (10.9%)
CT	**None (928; 3.9%)**	**Right (563; 2.4%)**	**Left (536; 2.3%)**	**One (415; 1.8%)**	Normal (372; 1.6%)	2,814 (12.0%)
MRI	**None (1,110; 3.5%)**	**Right (669; 2.1%)**	**Left (646; 2.0%)**	**One (589; 1.9%)**	Normal (484; 1.5%)	3,498 (11.0%)
Ultrasound	**None (32; 10.4%)**	Fluid (7; 2.3%)	**Right (5; 1.6%)**	**One (5; 1.6%)**	Bilateral (3; 1.0%)	52 (16.9%)
Radiography / Fluoroscopy	**None (45; 4.6%)**	**Right (15; 1.5%)**	**Left (15; 1.5%)**	Proximal (15; 1.5%)	**One (14; 1.4%)**	104 (10,5%)
Overall	**None (2,180; 3.6%)**	**Right (1,336; 2.2%)**	**Left (1,268; 2.1%)**	**One (1,054; 1.8%)**	Normal (876; 1.5%)	6,714 (11,2%)

Table 6 Overview of the top 5 most frequently deleted words from the impression section of analyzed radiology reports. Numbers in brackets represent count of deletions and corresponding fraction of total deletions in the respective subspecialty section or imaging modality. The last row represents results for all analyzed reports overall. The four most frequently deleted words overall (one, none, left, right) are marked in bold.

Section or Modality	Top 5 Most Frequently Deleted Words from Impression Section of Reports					
	1st word (N; % of total)	2nd word (N; % of total)	3rd word (N; % of total)	4th word (N; % of total)	5th word (N; % of total)	Sum N (% of total)
Body imaging	**One (544; 3.5%)**	**None (424; 2.8%)**	**Left (172; 1.1%)**	**Right (156; 1.0%)**	Recommended (115; 0.8%)	1,411 (9.2%)
Cardiothoracic imaging	**One (449; 3.1%)**	**None (353; 2.5%)**	**Right (218; 1.5%)**	**Left (184; 1.3%)**	Most likely (146; 1.0%)	1,350 (9.4%)
Musculoskeletal	**None (294; 2.5%)**	**One (263; 2.2%)**	**Right (158; 1.3%)**	**Left (135; 1.1%)**	As well as (134; 1.1%)	984 (8.2%)
Neuroradiology	**None (1,313; 3.5%)**	**One (1,185; 3.2%)**	**Right (666; 1.8%)**	**Left (631; 1.7%)**	Lesion (282; 0.8%)	4,077 (11.0%)
Breast Imaging	Recommended (125; 11.0%)	**None (62; 5.5%)**	Suspicious (51; 4.5%)	Follow Up (48; 4.2%)	Change (46; 4.1%)	332 (29.3%)
Nuclear medicine	**One (171; 3.0%)**	**Left (89; 1.6%)**	**Right (82; 1.4%)**	**None (67; 1.2%)**	Not (56; 1.0%)	465 (8.2%)
CT	**One (1,380; 3.0%)**	**None (1,292; 2.8%)**	**Right (727; 1.6%)**	**Left (671; 1.5%)**	As well as (335; 0.7%)	4,405 (9.6%)
MRI	**None (949; 3.2%)**	**One (927; 3.1%)**	**Right (434; 1.5%)**	**Left (421; 1.4%)**	Lesion (289; 1.0%)	3,020 (10.2%)
Ultrasound	**None (92; 4.9%)**	**One (88; 4.7%)**	Exam (57; 3.0%)	Unremarkable (38; 3.0%)	Free (32; 1.7%)	307 (17.3%)
Radiography / Fluoroscopy	**None (50; 3.1%)**	**One (47; 2.9%)**	Central (29; 1.8%)	**Right (22; 1.3%)**	As well as (20; 1.2%)	168 (10.3%)
Overall	**One (2,639; 3.1%)**	**None (2,513; 2.9%)**	**Right (1,294; 1.5%)**	**Left (1,218; 1.4%)**	As well as (632; 0.7%)	8,296 (9.6%)
